# Effects and Interaction of Meteorological Parameters on Influenza Incidence During 2010–2019 in Lanzhou, China

**DOI:** 10.3389/fpubh.2022.833710

**Published:** 2022-02-22

**Authors:** Jinyu Wang, Ling Zhang, Ruoyi Lei, Pu Li, Sheng Li

**Affiliations:** ^1^School of Basic Medical Sciences, Lanzhou University, Lanzhou, China; ^2^Institute of Occupational Health and Environmental Health, School of Public Health, Lanzhou University, Lanzhou, China; ^3^The Second People's Hospital of Baiyin, Baiyin, China; ^4^The First People's Hospital of Lanzhou, Lanzhou, China

**Keywords:** influenza, meteorology, distributed lag non-linear model, interaction, seasonally

## Abstract

**Background:**

Influenza is a seasonal infectious disease, and meteorological parameters critically influence the incidence of influenza. However, the meteorological parameters linked to influenza occurrence in semi-arid areas are not studied in detail. This study aimed to clarify the impact of meteorological parameters on influenza incidence during 2010–2019 in Lanzhou. The results are expected to facilitate the optimization of influenza-related public health policies by the local healthcare departments.

**Methods:**

Descriptive data related to influenza incidence and meteorology during 2010–2019 in Lanzhou were analyzed. The exposure-response relationship between the risk of influenza occurrence and meteorological parameters was explored according to the distributed lag no-linear model (DLNM) with Poisson distribution. The response surface model and stratified model were used to estimate the interactive effect between relative humidity (RH) and other meteorological parameters on influenza incidence.

**Results:**

A total of 6701 cases of influenza were reported during 2010–2019. DLNM results showed that the risk of influenza would gradually increase as the weekly mean average ambient temperature (AT), RH, and absolute humidity (AH) decrease at lag 3 weeks when they were lower than 12.16°C, 51.38%, and 5.24 g/m^3^, respectively. The low Tem (at 5th percentile, P_5_) had the greatest effect on influenza incidence; the greatest estimated relative risk (RR) was 4.54 (95%CI: 3.19–6.46) at cumulative lag 2 weeks. The largest estimates of RRs for low RH (P_5_) and AH (P_5_) were 4.81 (95%CI: 3.82–6.05) and 4.17 (95%CI: 3.30–5.28) at cumulative lag 3 weeks, respectively. An increase in AT by 1°C led to an estimates of percent change (95%CI) of 3.12% (−4.75% to −1.46%) decrease in the weekly influenza case counts in a low RH environment. In addition, RH showed significant interaction with AT and AP on influenza incidence but not with wind speed.

**Conclusion:**

This study indicated that low AT, low humidity (RH and AH), and high air pressure (AP) increased the risk of influenza. Moreover, the interactive effect of low RH with low AT and high AP can aggravate the incidence of influenza.

## Introduction

Influenza is an acute respiratory disease caused by the influenza virus, which belongs to category C infectious diseases in China. The influenza virus easily mutates and spreads mainly through aerosol droplets and contact (1, 2). Therefore, influenza infections can transform into outbreaks or epidemics. According to the World Health Organization, approximately 290,000–650,000 patients died due to respiratory tract infection, and 3–5 million people develop serious related diseases each year (3). Considering that the influenza virus is highly infectious and can cause severe morbidity, the burden of influenza cannot be neglected. Although China has established an influenza surveillance network, the latest research suggests that an average of 88,100 people die each year due to influenza-related respiratory diseases across the country (4, 5). It is essential to comprehend the epidemiological characteristics of influenza to reduce the burden of this disease. Because of global warming, the influence of meteorological parameters on the spread and incidence of influenza has become more critical. Seasonal changes in the incidence of respiratory diseases especially influenza reveal that the peak time of their occurrence is during winter and spring (6–9). The onset of influenza is sensitive to the climate, particularly temperature and humidity, giving rise to influenza seasonality in temperate regions. Low temperature significantly increases the incidence of influenza (10, 11). A study suggested that low humidity contributed to the spread of influenza in Shenyang, China (12). However, the relationship between influenza incidence and meteorological parameters such as air pressure (AP) and wind has yet to be clarified. Furthermore, it is difficult to hold a certain factor responsible for the occurrence of the disease because the effects of meteorological parameters are comprehensive and interdependent.

Lanzhou, located in northwestern China, experiences low precipitation, strong evaporation, and relatively lower temperature and is considered a typical arid city with a temperate semi-arid climate (13). The association between meteorological parameters and influenza incidence in semi-arid areas has rarely been reported. Therefore, this study was conducted to analyze the association between meteorological parameters and influenza in Lanzhou and to evaluate the interactions of RH with other meteorological parameters during 2010–2019. The results are expected to assist in the prevention and control of influenza infections, thereby reducing the burden of influenza in the future.

## Materials and Methods

### Data Collection

Data related to influenza cases were obtained from the Lanzhou Center for Disease Control and Prevention (CDC) legally reported infectious disease database from January 01, 2010 to December 31, 2019. The descriptive data included gender, age, diagnosis date, etc. Meteorological data were obtained from the daily report of Lanzhou Meteorological Bureau, including daily average ambient temperature (AT), RH, AP, Sunshine hours (SH), and wind speed (WS). Several demographic studies (14–17) have reported a significant relationship between the spread of influenza and absolute humidity (AH); therefore, this parameter was also included in the analysis.

### Calculation of AH

AH is the weight of water content per unit volume of gas, usually expressed as vapor pressure (VP) in g/m^3^. The ideal gas law (1) was combined with the Clausius–Clapeyron relationship (18) to calculate the saturated VP Es(T) (mb) from daily temperature (2) and then included the relative humidity (RH) (3) to derive the AH (19, 20) as shown below:


(1) $$\begin{array}{lll} \rho v & = & 1000\times \frac{v}{G_{v}T} \end{array}$$

(2) $$\begin{array}{lll} E_{S}\left(T\right) & = & E_{S}\left(T_{0}\right)\times EXP\left[\frac{L}{G_{v}}\left(\frac{1}{T_{0}}-\frac{1}{T}\right)\right] \end{array}$$

(3) $$\begin{array}{lll} VP & = & 100\times E_{s}\left(T\right)\times \frac{RH}{100} \end{array}$$

Where *G*_*v*_ is the gas constant of water vapor [461.53 J/(kg·K)]; *v* is the VP; *T* is the daily AT (K); *E*_*S*_(*T*) is the saturated VP; *T*_0_ is the reference temperature (273.15 K); *L* is the standard latent heat of evaporation for 1 kg of water (2,257 kJ/kg).

### Case Definition

The influenza case was defined as a person with a sudden onset of fever (≥38°C), chills, cough and/ or sore throat, a generalized feeling of weakness and pain in the muscles, together with varying degrees of soreness in the head and abdomen. Influenza cases should meet the standard diagnostic criteria for influenza (WS 285-2008) from the National Health Commission of the People's Republic of China. All these influenza cases were diagnosed and confirmed by a positive pathogenic test of the influenza virus. Once diagnosed, each case must be reported to the National Information System for Disease Control and Prevention immediately and we thus obtained the daily incidence data of influenza from this reporting system.

### Statistical Analysis

Considering the over-dispersion of the daily incidence of influenza, a weekly time-series database of influenza incidence and meteorological parameters was established for the period of 2010–2019 in Lanzhou. First, a descriptive analysis was performed on the collected data. Then, the Spearman correlation analysis was used to explore the correlation between meteorological parameters and weekly influenza case counts. The distributed lag non-linear model (DLNM) with Poisson distribution was applied to explore the effects of meteorological parameters on influenza incidence, which included weekly mean average AT, RH, AP, WS, AH, and weekly cumulative SH. Next, the response surface model and stratified model were used to indicate the interaction effects between RH and other meteorological parameters (AT, AP, and WS) on influenza incidence. Finally, a sensitivity analysis was performed by changing the degree of freedom of the penalized smoothing spline function from 3 to 8 for Tem and humidity (AH and RH) and either controlling or not controlling the autocorrelation.

The DLNM model was established for each variable including AT, AH, RH, AP, SH, and WS, while controlling for long-term trend and seasonality by using a time-stratified method (employing simple indicator variables) (21, 22). Considering the delayed impact of infection and morbidity (23), the maximum lag period was determined to be 3 weeks to estimate the cumulative effect (relative risk, RR) of the meteorological parameters on influenza incidence. By using the median level of each meteorological parameter as a reference, the cumulative RR and 95% confidence interval (CI) were calculated to determine the extreme effects of low and high levels in the 5th and 95th percentiles (P_5_ and P_95_) in the model. For example, the effect of low RH on influenza incidence was analyzed by comparing the 5th percentile with the median level. The main model (24) was as follows:


(4) $$\begin{array}{l} \log[E(Y_{i})]=\alpha +cb(X_{i,l})+\sum_{}^{}ns(Z_{i,t},df)+strata\\ \;+\log(Y_{i-1}) \end{array}$$

Where i is the week of the observation; *Y*_*i*_ is the observed weekly case counts in Lanzhou during week *i*; *E*(*Y*_*i*_) is the expected number of influenza case counts during week *i*; α is the intercept; *cb*() represents the two-dimensional model used to fit the non-linearity and lag weeks of meteorological parameters by using cross-basis function; *X*_*i, l*_ represents the meteorological parameters during week *i*, indicates the weekly mean AT (*AT*_*i, l*_), mean RH (*RH*_*i, l*_), mean AH (*AH*_*i, l*_), mean AP (*AP*_*i, l*_), mean WS (*WS*_*i, l*_), and SH (*SH*_*i, l*_), respectively. *Z*_*i, t*_ refers to other meteorological parameters except for *X*_*i, l*_; the strata variable represents an indicator to control the long-term trend and seasonality for the combination of year and month; log(*Y*_*i*−1_) represents the number of influenza cases in the previous week to control the autocorrelation; the degree of freedom (*df*) of the natural spline smoothing function in the formula was selected according to the Akaike's information criteria. Concerning the collinearity, two variables having a high correlation (Spearman correlation coefficient >0.7) were not included in the same model.

Considering the characteristics of the temperate semi-arid climate in Lanzhou, the response surface model and stratified model (25) were established to fit the interaction effect of RH with other meteorological parameters on influenza incidence through three-dimensional maps. In the stratified model, the 5th and 95th percentiles were used as tangents for the RH to be divided into two-categorical variables, which were divided into two levels: “low” and “high.” Low-RH referred to the case when RH was less than the 5th percentile, and High-RH referred to the case when the RH was higher than the 95th percentile. The model is shown in Equations (5) and (6):


(5) $$\begin{array}{l} \log[E(Y_{i})]=\alpha +tp(RH,X_{i})+ns\left(weather\right)+strata\\ \;+\log(Y_{i-1}) \end{array}$$

(6) $$\begin{array}{l} \log\left[E\left(Y_{i}\right)\right]=\beta_{1}X_{i}+\beta_{2}RH_{b}+\beta_{3}X_{i}:RH_{b}+ns(weather)\\ \;+strata+\log\left(Y_{i-1}\right) \end{array}$$

Where *tp*() represents the response surface function; *X*_*i*_ represents the meteorological parameters (AT, SH, and WS) that interact with RH; *weather* represents other meteorological parameters except *X*_*i*_; *RH*_*b*_ represents two-categorical variables of RH; β_3_ is the interaction effect of RH with other meteorological parameters on influenza case counts; other variables are the same as those in foregoing model.

All analyses were conducted by using the “mgcv” and “dlnm” packages in R4.0.0 in this study. Results were considered statistically significant when *P* < 0.05.

## Results

### Descriptive Data Related to Weekly Influenza Case Counts and Meteorological Data

**Figure 1:**
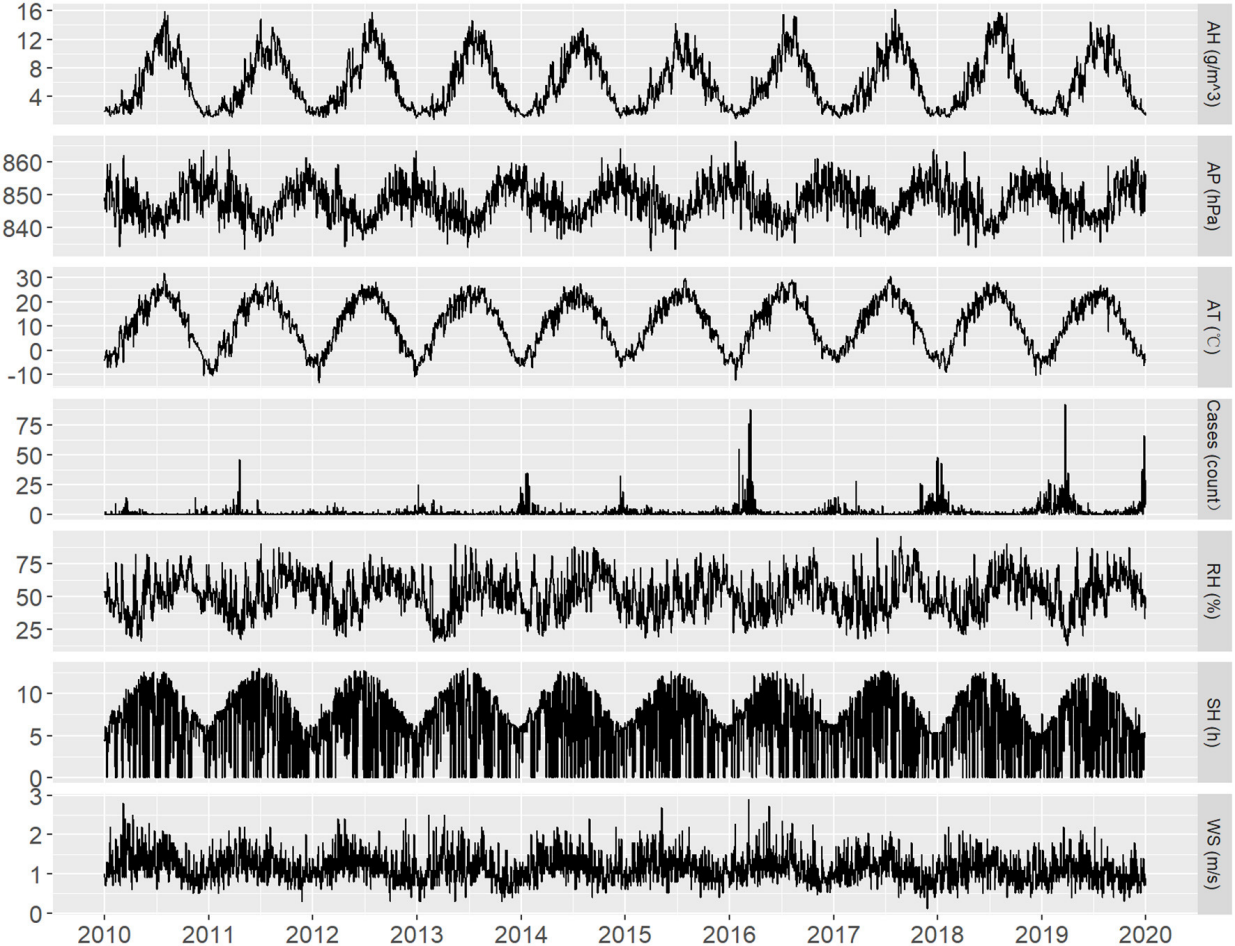
Time-series distribution of daily influenza case counts and daily meteorological parameters in Lanzhou, China during 2010–2019.

During the study period (2010–2019), a total of 6,701 cases of influenza were reported in Lanzhou, of which 3,905 were in males and 3,374 were in females (sex ratio 1.13). Among the patients, 53.93% were children under the age of 14 years. Table 1 shows the summary statistics of the meteorological variables. The mean values of AT, RH, AH, AP, SH, and WS were respectively 11.12°C, 51.21%, 6.01 g/m^3^, 848.01 hPa, 43.42 h, and 1.15 m/s. Table 2 shows the correlation between influenza case counts and meteorological parameters. All meteorological parameters including AT, RH, WS, AH, and SH were negatively correlated with influenza incidence (*P* < 0.05), while AP was positively correlated with influenza incidence. The correlation of AT and AH with influence incidence was stronger (r_AT_ = −0.53 and r_AH_ = −0.55, respectively) than that of others. The time-series distribution of weekly meteorological parameters and influenza case counts from 2010 to 2019 demonstrated a clear seasonal pattern (Figure 1).

**Table 1:** Basic information related to influenza case counts and meteorological parameters in Lanzhou, China during 2010–2019.

**Variable**	**Mean**	**S.D**.	**Min**	**Percentiles**			**Max**
				**25**	**50**	**75**	
AT (°C)	11.12	9.88	−8.84	1.72	12.76	19.93	29.86
AP (hPa)	848.01	4.38	838.47	844.31	848.38	851.40	858.26
RH (%)	51.21	12.28	18.39	42.92	52.04	60.43	87.00
WS (m/s)	1.15	0.22	0.56	0.99	1.14	1.30	1.76
AH (g/m^3^)	6.01	3.62	1.24	2.51	5.26	8.96	14.40
SH (h)	43.42	14.45	1.30	32.70	43.30	53.95	83.20
Cases (counts)	12.79	25.07	0.00	2.00	5.00	12.00	311.00

*S.D., standard deviation; Min, minimum; Max, maximum. AT, RH, AH, AP, WS, and SH represent, respectively, weekly mean average ambient temperature, mean relative humidity, mean absolute humidity, mean air pressure, mean wind speed, and weekly cumulative sunshine hours, respectively. “Cases” represents weekly influenza case counts*.

**Table 2:** Spearman correlation between influenza case counts and meteorological parameters in Lanzhou, China during 2010–2019.

**Variable**	**Cases**	**AT**	**RH**	**WS**	**AH**	**AP**	**SH**
Cases	1.00						
AT	−0.53^*^	1.00					
RH	−0.21^*^	0.03	1.00				
WS	−0.24^*^	0.41^*^	−0.41^*^	1.00			
AH	−0.55^*^	0.92^*^	0.41^*^	0.23^*^	1.00		
AP	0.38^*^	−0.79^*^	0.16^*^	−0.52^*^	0.16^*^	1.00	
SH	−0.20^*^	0.47^*^	−0.59^*^	0.46^*^	0.20^*^	−0.44^*^	1.00

“*”Represents P <0.05. AT, RH, AH, AP, WS, and SH represent, respectively, weekly mean average ambient temperature, mean relative humidity, mean absolute humidity, mean air pressure, mean wind speed, and weekly cumulative sunshine hours, respectively. “Cases” represents weekly influenza case counts.

### Lag Effect of Meteorological Parameters on Influenza Incidence

**Figure 2:**
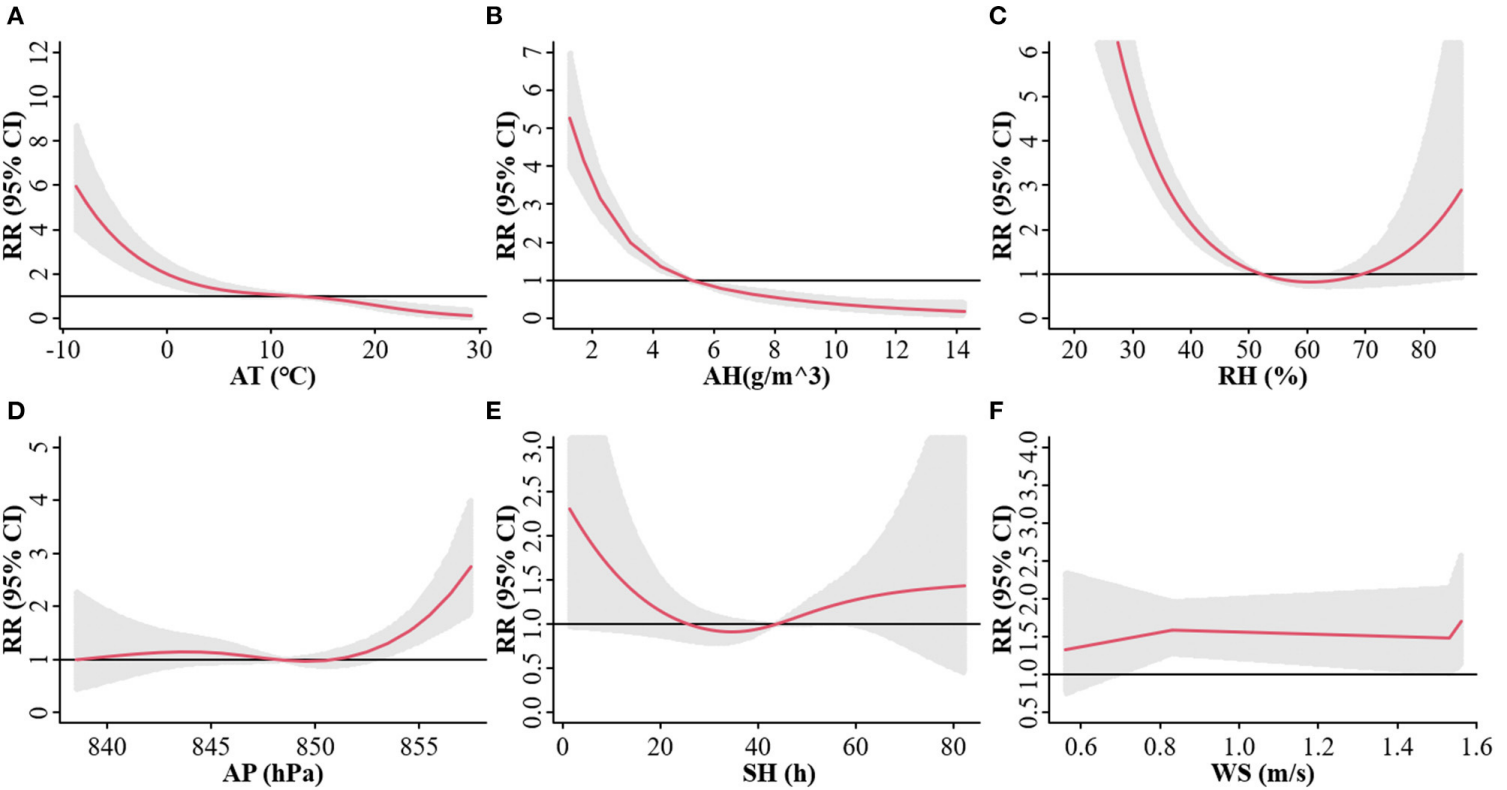
Exposure-response relationship between the risk of influenza and AT **(A)**, AH **(B)**, RH **(C)**, AP **(D)**, SH **(E)**, and WS **(F)** at a three-week lag in Lanzhou, China during 2010–2019. The red line represents the cumulative relative risk (RR) of influenza, and the gray shaded region represents the 95% confidence interval (CI).

The correlation between meteorological parameters and weekly influenza case counts was estimated by the cumulative RR after a lag of 3 weeks from the DLNM model (Figure 2). After controlling for the long-term trend and seasonality, the median of each meteorological parameter was taken as reference, and an approximately “L”-shaped correlation was observed between AT, RH, and AH and the risk of influenza. The risk of influenza gradually increased as the AT, RH, and AH decreased, when they were lower than 12.16°C, 51.38%, and 5.24 g/m^3^, respectively. A “J”-shaped correlation was observed between AP and the risk of influenza. When AP was higher than 853.47 hPa, the risk of influenza rapidly increased as the AP increased. Medium WS increased the risk of influenza. Sensitivity analysis displayed steady results which were insensitive to the specifications of the parameters (Supplementary Figures 1, 2).

**Figure 3:**
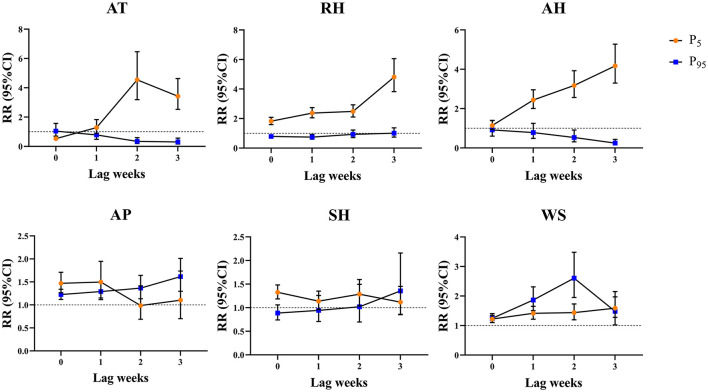
Cumulative lag effect of meteorological parameters on the onset of influenza under extreme conditions at different lag weeks in Lanzhou, China during 2010–2019. P_5_ represents the 5th percentile. P_95_ represents the 95th percentile.

Based on the above results, we analyzed the lag effect of various meteorological parameters on the onset of influenza at different lag days under extreme conditions fitted by the DLNM model, such in cold (< -4.64°C, P_5_ of AT) and hot (>24.49°C, P_95_ of AT) conditions (Figure 3). With various lag weeks, a significant effect of cold and hot weathers was observed on influenza incidence during 1–3 lag weeks. Low AT (P_5_) exhibited the largest estimated effect at lag 2 weeks, and the cumulative RR was 4.54 (95%CI: 3.19–6.46). Significant effects of dry RH and dry AH were observed during 0–3 lag weeks. Dry RH (P_5_) and dry AH (P_5_) exhibited the largest estimated effect at lag 3 weeks, and the cumulative RRs were 4.81 (95%CI: 3.82–6.05) and 4.17 (95%CI: 3.30–5.28), respectively. The relative wet effect [RH (P_95_) and dry AH (P_95_)] reduced the risk of influenza. The extreme effect of AP and WS was not significant, and a dangerous effect was observed at every lag week. The significant extreme effect of SH on influenza incidence showed that lesser SH increased the risk of influenza.

### Interaction Effect of Meteorological Parameters on Influenza Incidence

**Figure 4:**
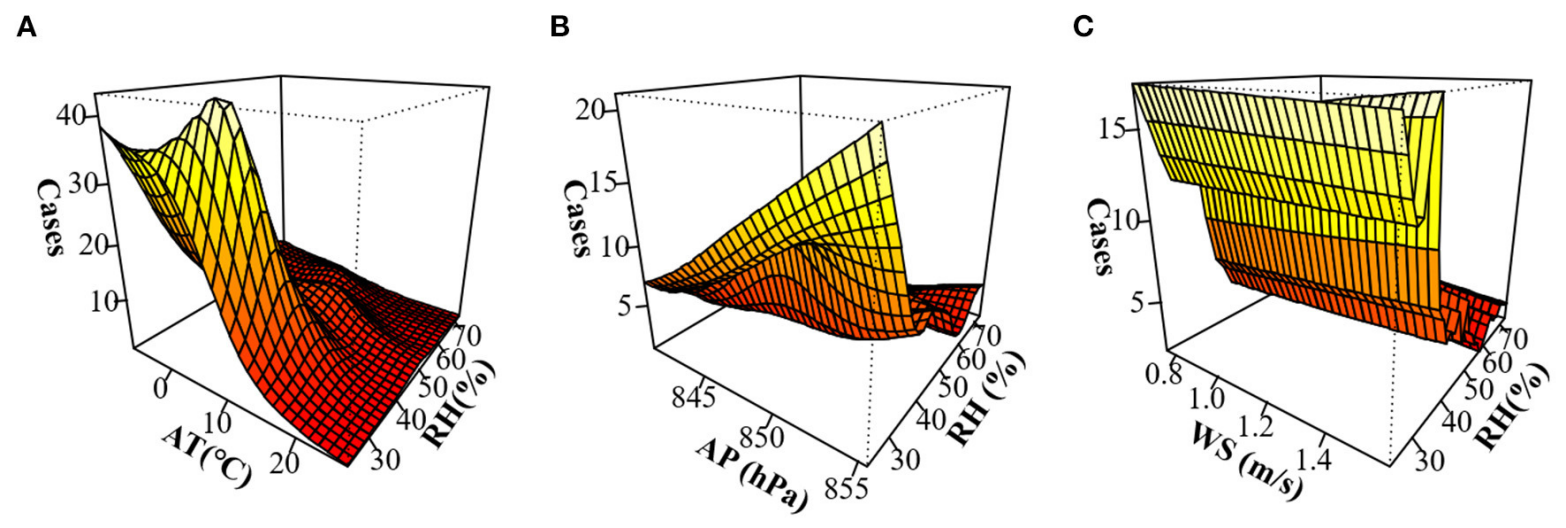
Three-dimensional map of the interaction effect of relative humidity (RH) with other meteorological parameters on influenza incidence in Lanzhou, China during 2010–2019 after a lag of 3 weeks. **(A)** Interaction effect of RH with AT. **(B)** Interaction effect of RH with AP. **(C)** Interaction effect of RH with WS.

Because Lanzhou is a typical temperate semi-arid climate, the interaction effect of RH with other meteorological parameters on influenza incidence was studied. The hierarchical model analysis is shown in Table 3. At lag 3 weeks, there was an interaction effect of Low-RH with AT, AP, and WS (*P* < 0.05) on influenza incidence, while the interaction effect of High-RH was not significant. An increase in AT by 1°C led to an estimates of percent change (95%CI) of 3.12% (−4.75 to −1.46%) decrease in the weekly influenza case counts with in the Low-RH environment. Influenza case counts were affected by various meteorological parameters and the interaction between various meteorological parameters. The interactive model also showed that there was an obvious interaction effect of RH with AT and AP on influenza incidence (Figure 4). In three-dimensional analysis, the strongest interaction was observed under conditions of low RH, low AT, and high AP.

**Table 3:** Estimates of percent change (95% CI) in weekly influenza cases associated with a 1-unit increase in other meteorological parameters stratified by RH.

	**Low-RH**	**High-RH**
AT	−3.12% (−4.75%, −1.46%)^*^	0.42% (−2.38%, 3.30%)
AP	−5.42% (−7.99%, −2.22%)^*^	−1.03% (−1.03%, 3.99%)
WS	97.80% (29.78%, 201.46%)^*^	104.90% (−42.85%, 634.27%)

“*”represents significance at the level of 0.05. Taking the 5th and 95th percentiles as tangent points of the binary categorical variables, relative humidity (RH) was divided into two levels of “low” and “high.” When RH was less than the 5th percentile, the environment was defined as Low-RH. When RH was greater than the 95th percentile, the environment was defined as High-RH.

## Discussion

The antigenic drift and transfer of the influenza virus are constantly changing, imposing huge economic losses and disease burdens to the public on a global scale (5, 26–28). In this study, the relationship between meteorological parameters and influenza incidence was analyzed in a semi-arid area (Lanzhou) for the first time. Low AT, low humidity (AH and RH), and high AP could accelerate the spread of influenza with obvious lag effects. Moreover, in this dry area, the interaction effect of low RH with low AT and high AP on influenza incidence was significant.

From January 1, 2010 to December 31, 2019, a total of 6701 influenza cases were reported in Lanzhou. Children under the age of 14 years were a high-risk group, accounting for 53.93% of the total influenza patient population, consistent with other studies (9, 29). The seasonality of change trend demonstrated in previous studies (30–32) has indicated that the incidence of influenza peaks in winter and spring, particularly when the AT is the lowest. In an animal experiment, the airborne transmission of influenza virus was enhanced when guinea pigs were housed at a low AT (5°C), while at a high AT (30°C), the spread was interrupted under all conditions of RH (33). The reasons for low AT facilitating the spread of influenza may be as follows: (1) Influenza virus can survive at 0–4°C for several weeks and for a long time below −70°C or after freeze-drying, but its infectivity is quickly lost at room temperature. The average temperature during winter in Lanzhou is close to its optimum growth temperature, making it easier to spread and infect. Some studies also indicated that the influenza virus envelope was more complete and the survival time was longer at low temperatures than at high temperatures (34, 35). (2) Children under the age of 14 years were at high risk for influenza, probably because they had poor awareness of disease prevention, habit of hygiene, and immune system, making this population group more susceptible to be infected in cold weathers (36). (3) People spend more time indoors during cold weather, which could lead to higher infection rates and epidemics (37).

In this study, low humidity (RH and AH) was more beneficial to the activity of influenza, and a significant dry effect of RH and AH was observed during 0–3 lag weeks. The results of this study were consistent with those of studies on the impact of humidity on influenza (12, 38, 39). It is suggested that low humidity improves the survival stability of the virus when the salt content of droplets in winter allows the virus to maintain high vitality at low RH (<50%), and the viability of influenza virus increased with decreasing RH (40). Low humidity exposure impaired host responses to infection, resulting in higher viral burden such as decreasing mucociliary clearance in mouse trachea (41). However, conflicting views have been reported regarding the impact of RH on influenza. The findings of the current study on RH were not in line with those reported by studies in Poland, Zhejiang, and Chongqing, in which the influenza incidence was moderately positively correlated with RH, and higher RH could increase the risk of influenza incidence (42–44). The difference in the results may be because of the difference in the latitude, climate type, demographic characteristics of the study area, and the statistical methods and models used. Therefore, the reported associations related to influenza incidence are specific to the regions.

The interaction model in this study showed the interaction effect of RH with other meteorological parameters. The condition of low RH with low AT could increase the influenza case counts. Recent laboratory and epidemiological evidence have confirmed that influenza virus transmission depends on RH and AT, and the onset of influenza exhibits a synchronous change pattern with cold and dry climate conditions, revealing that low RH with low AT increases the risk of influenza epidemic (33, 45, 46). Temperature and humidity were the lowest when influenza was most active during winter and spring. Therefore, temperature and humidity play important roles in the spreading of influenza. Previous studies had been carried out in temperate or humid regions, but the present study shows how temperature and humidity influence the incidence of influenza in an arid region.

Besides the commonly studied parameters of temperature and humidity, other meteorological parameters (WS, SH, and AP) were also analyzed in this study. The risk of influenza rapidly increased as AP increased, when the AP was >853.47 hPa, and the interactive effect of low RH with high AP could increase the incidence of influenza, revealing a synergistic effect. A study similarly reported that under high pressure, the number of influenza cases increased when AP was >1,005 hPa (47). It is well-known that high AP usually accompany cold and dry weather, which promote indoor activities and more communication among people, this may increase the spread risk of influenza infection. Besides, the cold and dry weather would also make the nasal mucosa vulnerable to be cracked, which may increase the risk of influenza invasion.

### Limitations

This study deepens the understanding of the effects of meteorological parameters on influenza incidence by using weekly data in a temperate semi-arid region. However, some limitations must be acknowledged. First, individuals most often overlook influenza and choose to isolate themselves at home due to its relatively mild symptoms, increasing the chances of a missed diagnosis. Second, the influenza virus was not classified in this study, and the impact of meteorological parameters may be different on different types of influenza viruses. Third, sandstorms, smog, and other extreme weather events in Lanzhou during peak times and air pollutants can also affect influenza seasonality (10, 42, 48–50). The number of days of extreme weather are increasing each year due to global climate change, which may pose more threats to public health. Finally, this is an ecological study, so the ecological bias could not be avoided. But it at least provide a hypothesis and indicate the significant effect of environmental factors on the influenza infection, which may be important for the government to place suitable medical cares against influenza in different weathers.

## Conclusions

Low AT, low humidity (RH and AH), and high AP increased the risk of influenza. Moreover, the interaction effect of low RH with low AT and high AP can aggravate the incidence of influenza. Considering these significant effects of meteorological parameters, relevant government departments could actively implement appropriate measures to optimize influenza-related public health policies such as monitoring the mutations of influenza virus in a timely manner and providing increased vaccine coverage during the cold and dry season.

## Data Availability Statement

The original contributions presented in the study are included in the article/Supplementary Material, further inquiries can be directed to the corresponding author/s.

## Author Contributions

JW: methodology, software, data curation, and writing—original draft preparation. LZ: methodology, software, visualization, and writing—original draft preparation. RL: methodology and data curation. PL: investigation. SL: conceptualization, supervision, formal analysis, and writing—reviewing and editing. All authors contributed to the article and approved the submitted version.

## Funding

This work was supported by the Natural Science Foundation of Gansu Province (17JR5RA347).

## Conflict of Interest

The authors declare that the research was conducted in the absence of any commercial or financial relationships that could be construed as a potential conflict of interest.

## Publisher's Note

All claims expressed in this article are solely those of the authors and do not necessarily represent those of their affiliated organizations, or those of the publisher, the editors and the reviewers. Any product that may be evaluated in this article, or claim that may be made by its manufacturer, is not guaranteed or endorsed by the publisher.
