# Supplementary material for: Effects and Interaction of Meteorological Parameters on Influenza Incidence During 2010–2019 in Lanzhou, China
Source: Front Public Health. 2022 Feb 22;10:833710. doi: 10.3389/fpubh.2022.833710 (PMC8902077; doi:10.3389/fpubh.2022.833710)
Supplement: Supplementary file 1 [file Table_1.DOCX]

Supplementary Material

# Supplementary Figures


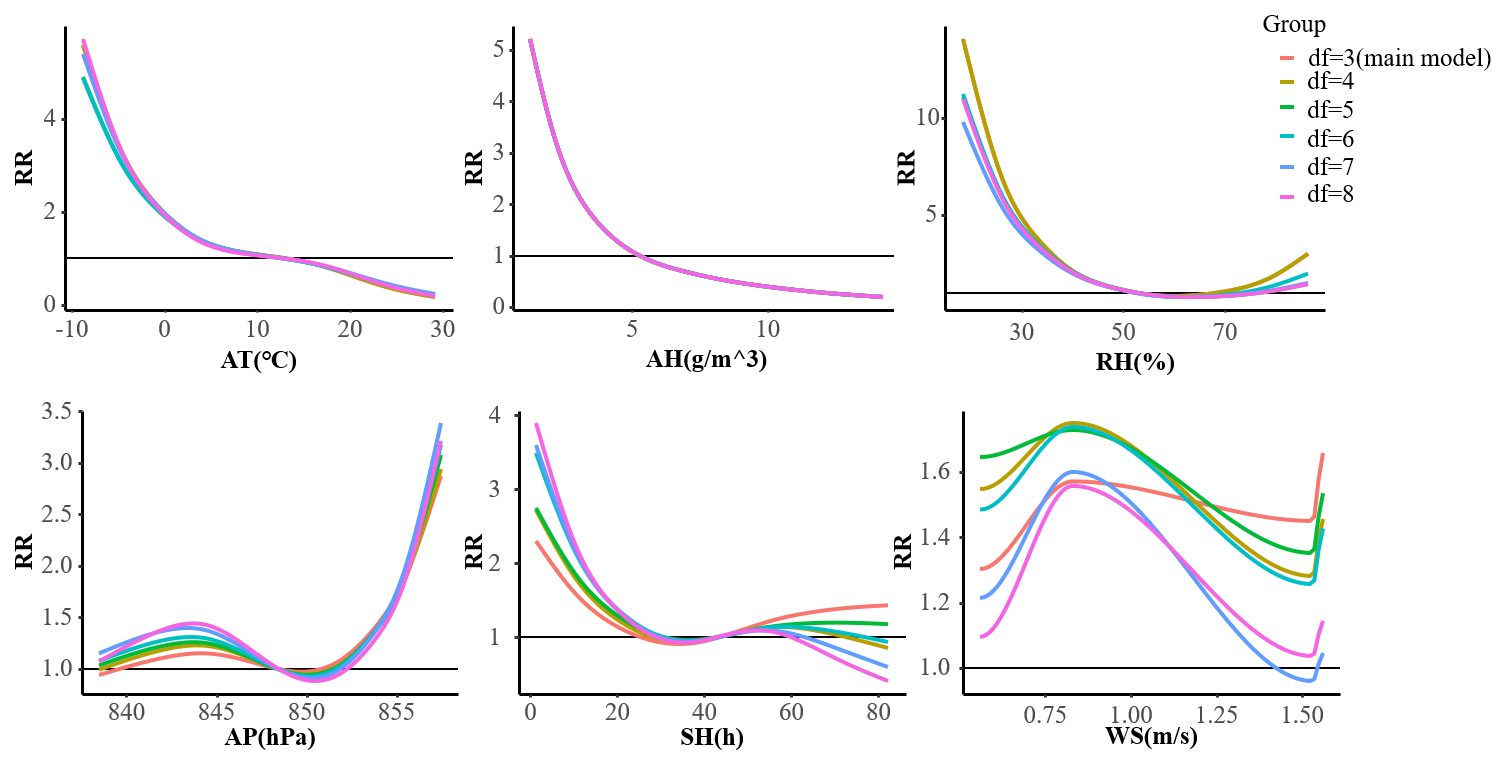


**Supplementary Figure 1** **|** Pooled estimates of meteorological parameters on influenza when changing the degree of freedom of the penalized smoothing spline function from 3 to 8 for Tem and humidity (AH and RH).


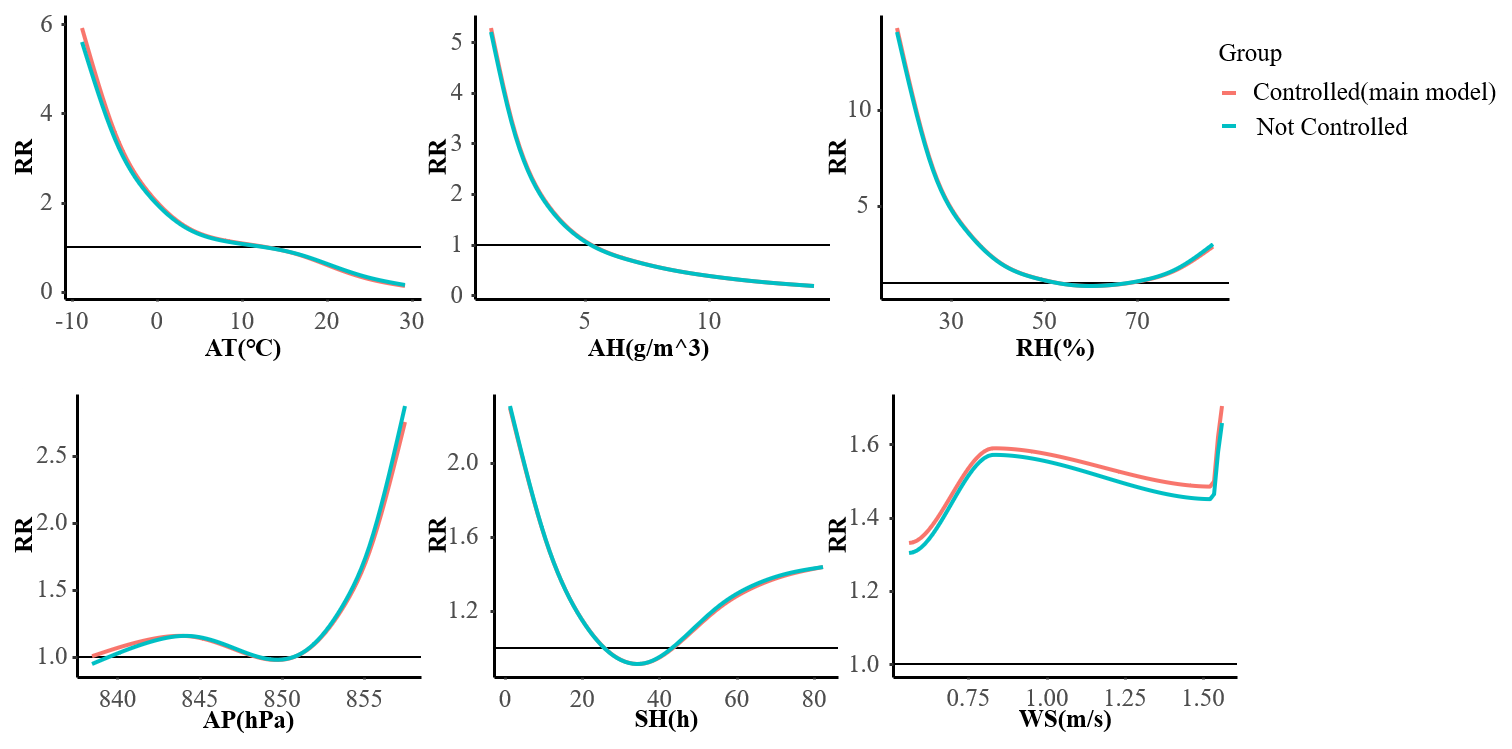


**Supplementary Figure 2** **|** Pooled estimates of meteorological parameters on influenza when either controlling or not controlling the autocorrelation. The two lines are nearly overlapped.
